# A Context-Aware Smartphone-Based 3D Indoor Positioning Using Pedestrian Dead Reckoning

**DOI:** 10.3390/s22249968

**Published:** 2022-12-17

**Authors:** Boshra Khalili, Rahim Ali Abbaspour, Alireza Chehreghan, Nahid Vesali

**Affiliations:** 1 School of Surveying and Geospatial Engineering, College of Engineering, University of Tehran, Tehran P.O. Box 14155-6619, Iran; 2 Mining Engineering Faculty, Sahand University of Technology, Tabriz P.O. Box 51335-1996, Iran; 3 Department of Engineering Leadership and Program Management, School of Engineering, The Citadel, Charleston, SC 29409, USA

**Keywords:** indoor positioning, PDR, smartphone sensors, context-aware, machine learning

## Abstract

The rise in location-based service (LBS) applications has increased the need for indoor positioning. Various methods are available for indoor positioning, among which pedestrian dead reckoning (PDR) requires no infrastructure. However, with this method, cumulative error increases over time. Moreover, the robustness of the PDR positioning depends on different pedestrian activities, walking speeds and pedestrian characteristics. This paper proposes the adaptive PDR method to overcome these problems by recognizing various phone-carrying modes, including texting, calling and swinging, as well as different pedestrian activities, including ascending and descending stairs and walking. Different walking speeds are also distinguished. By detecting changes in speed during walking, PDR positioning remains accurate and robust despite speed variations. Each motion state is also studied separately based on gender. Using the proposed classification approach consisting of SVM and DTree algorithms, different motion states and walking speeds are identified with an overall accuracy of 97.03% for women and 97.67% for men. The step detection and step length estimation model parameters are also adjusted based on each walking speed, gender and motion state. The relative error values of distance estimation of the proposed method for texting, calling and swinging are 0.87%, 0.66% and 0.92% for women and 1.14%, 0.92% and 0.76% for men, respectively. Accelerometer, gyroscope and magnetometer data are integrated with a GDA filter for heading estimation. Furthermore, pressure sensor measurements are used to detect surface transmission between different floors of a building. Finally, for three phone-carrying modes, including texting, calling and swinging, the mean absolute positioning errors of the proposed method on a trajectory of 159.2 m in a multi-story building are, respectively, 1.28 m, 0.98 m and 1.29 m for women and 1.26 m, 1.17 m and 1.25 m for men.

## 1. Introduction

Indoor positioning systems have been studied as a means of guiding pedestrians around buildings, particularly in emergencies [1]. A variety of indoor positioning systems are currently available, including WLAN-based [2], Bluetooth low energy (BLE) [3,4], ultra-wideband (UWB) [5,6], Ultrasonic Based [7] and infrared [8,9]. In these methods, position errors do not accumulate over time. However, they require the development of a positioning infrastructure before navigation; hence in an unknown environment, they would be impossible to be used and would require a significant cost [10]. PDR is, rather, an infrastructure-free, effective method that utilizes the estimated pedestrians’ steps, step length and heading angle to determine the position [11]. Using PDR for positioning has several advantages; its main benefit is that it does not require additional infrastructure and uses body-mounted or smartphone-embedded inertial sensors such as a magnetometer, gyroscope and accelerometer for positioning [10,12]. However, this method also has its drawbacks. The major problem with PDR is the accumulated position error, which increases over time and arises from various parts of the method, such as step length estimation, step detection and heading estimation errors [10,13,14,15]. The second problem is that variations in motion states, including phone-carrying modes and pedestrian activities, walking speeds and characteristics of pedestrians compromise the robustness of its positioning [1,14,16,17]. Adjusting the parameters of different parts of PDR considering suitable pedestrian characteristics and motion states led to an improvement in the accuracy and robustness of PDR positioning. Step length estimation, which is a source of cumulative errors, plays a crucial role in the PDR method [18]. Step length is affected by height, which in turn depends on gender. According to the available data, the global average height for men is 171 cm while for women it is 159 cm (https://ourworldindata.org/human-height, accessed on 1 November 2022). This indicates on average that men are 12 cm taller than women. Therefore, gender is a reasonable parameter for considering two height categories. In addition, pedestrians’ step lengths vary due to their walking speed, because acceleration data differs across walking speeds. In the long and complicated paths in buildings, the pedestrian moves at different speeds depending on different situations. Thus, the positioning error can be decreased by considering the different walking speeds. Hence, considering varying pedestrian characteristics as well as detecting various motion states and walking speeds can improve the robustness of PDR positioning.

In this article, an adaptive PDR method is proposed to improve the robustness and accuracy of Three-dimensional positioning by adjusting its parameters based on different phone-carrying modes, pedestrian activities, walking speeds and individual characteristics. The proposed classification approach uses a combination of support vector machine (SVM) and decision tree (DTree) algorithms to recognize motion states. Additionally, the parameters of a step detection and step length estimation model are adjusted based on gender, the detected motion states and walking speeds. The main contributions of this research are as follows:

- PDR positioning is more adaptable when considering various phone-carrying modes, including texting, calling and swinging, as well as different pedestrian activities, including ascending and descending stairs and walking. This is because sensor data differ for different phone-carrying modes and pedestrian activities. The acceleration data also differ across walking speeds as walking can be classified as fast, medium and slow; thus, positioning accuracy is improved and adapted to changes in walking speed. This paper uses the DTree and SVM to identify various motion states and walking speeds. Using the proposed classification strategy, 15 combinations of five pedestrian activities and three phone-carrying modes are accurately distinguished.
- In addition to detecting different motion states and walking speeds, considering height and gender as effective parameters in estimating step length promotes distance estimation accuracy. By analyzing each motion state separately for women and men, PDR positioning is further adapted to diverse heights and genders, so the overall accuracy of positioning improves.
- After state detection, parameters of step counting and methods for step length estimation are separately adjusted for each pedestrian activity, phone-carrying mode, walking speed and gender to enhance the robustness and accuracy of PDR positioning.

The remainder of this paper is organized as follows: Section 2 discusses the literature. The overview of the implementation of the positioning system is presented in Section 3. In Section 4, the performance of the proposed method is empirically evaluated. Conclusions and future research directions are discussed in Section 5.

## 2. Related Work

Three-dimensional (3D) indoor positioning using the PDR method consists of various components such as step detection, step length estimation, heading determination and altitude determination. Several methods have been developed for step detection based on accelerometer sensors, such as threshold setting [19], peak detection [20] and correlation analysis [21]. The second component of the PDR is step length estimation. An accurate estimate of step length plays a critical role in the PDR system. To estimate step length, artificial neural networks (ANNs) [17,22,23,24] and empirical models [16] have generally been utilized by many researchers. Several investigations have used neural networks to improve the accuracy of step length estimation. This approach requires large data sets. In addition, the increased complexity and time consumption make it difficult to be used in smartphones and embedded systems [1]. However, several researchers have investigated empirical models. Ladetto [25] utilized an empirical model that combines the step frequency and variance of the sensor signal to estimate the step length. Weinberg [26] also used the quartic root of the difference between the maximum and minimum of *z*-axis acceleration. Kim and Jang [27] utilized the cubic root of the average acceleration magnitude to estimate the step length. These empirical models can achieve high accuracy under typical walking conditions. Therefore, empirical models should be adapted to various activities [1]. Moreover, the parameters of these empirical models should be adjusted according to different phone-carrying modes [16]. Movement habits should also be considered in setting parameters of empirical models. Since the movement habits of each person are derived based on age, gender, walking speed and height, the model’s parameters should be adjusted according to the abovementioned characteristic [28].

Heading determination, which is a source of cumulative errors, plays a crucial role in the PDR method. Using only a gyroscope for heading determination causes a larger cumulative error. Besides, the cumulative error increases with time [14]. Fusion filter algorithms, including complementary filters [11], Kalman filters [29], extended Kalman filters [30], unscented Kalman filters [31] and Madgwick filters [32] have been proposed to improve the heading accuracy. Numerous studies have also classified different phone-carrying modes, human activities and movement habits. Some researchers have used machine learning methods, such as SVM [1,33], K-nearest-neighbors (KNN) [33,34], DTree [33,35], naive Bayes [34,36], multilayer perceptron [16] and random decision forests [37]. Several researchers have also adopted deep learning methods, such as long-short-term memory (LSTM) [17,23,38,39], ANN [10], recurrent artificial neural networks (RNN) [40] and convolutional neural networks (CNN) [38,39].

Wang and Liu [33] used SVM and DTree to detect the combination of movement state and phone-carrying modes. A method based on principal component analysis [41] with global accelerations (PCA-GA) was also proposed for pedestrian heading estimation. Klein and Solaz [16] investigated the effect of phone-carrying modes on the accuracy of step length determination. They used the KNN, multilayer perceptron, SVM, gradient boosting and random forest algorithms to recognize four phone-carrying modes (swinging, talking, texting and in the pocket). The best accuracy of 95.4% was achieved by the gradient boosting algorithm. They also chose an appropriate parameter in the empirical model of step length estimation according to each phone-carrying mood. Gu and Khoshelham [18] proposed a model for step length estimation based on the stacked auto-encoders approach, which considered different walking speeds and phone-carrying modes and was adapted to different users’ characteristics. Xu and Xiong [10] used an ANN to recognize three phone poses. The peak detection method was implemented to count steps for various phone-carrying modes, while a neural network and differential GPS were used to estimate step lengths. A zero angular algorithm was proposed to correct the heading error caused by switching the smartphone carrying mode.

Wang and Ye [23] proposed a step length estimation method based on LSTM and de-noising auto-encoders, called tapeline. This method achieves good estimation accuracy, with a step-length error of 4.63% and a walking-distance error of 1.43% without relying on pre-collected databases when a pedestrian walks in complex environments (stairs, spiral stairs, or elevators) with motion patterns (fast walking, typical walking, slow walking, running, or jumping). The significant disadvantages of tapeline were that the LSTM network and noise reduction procedures involve significant processing overhead and consider only the texting smartphone carrying mode. Wang and Ye [42] proposed a smartphone mode recognition algorithm using a stacking regression model to effectively determine various smartphone carrying modes (calling, handheld, pocket, armband and swing), with an average recognition accuracy of 98.82%. The proposed method results in an error of 3.30% for step length estimation and 2.62% for walking distance estimation. Lu and Wu [43] designed a fuzzy controller based on the fuzzy logic algorithm to adaptively adjust the constant coefficient k in Weinberg’s nonlinear step length estimation (SLE) model at each detected step, which is measured based on each user’s different speed of walking. Ye and Li [38] designed and trained deep learning models via LSTM and CNN networks based on the tensor flow framework for pedestrian motion mode, smartphone posture and real-time comprehensive pedestrian activity recognition. Xia and Huang [39] introduced a combination of LSTM and CNN architecture for human activity recognition, with an accuracy of 95.78%, 95.85% and 92.63%, respectively, which was validated using three public datasets, i.e., UCI, WISDM and Opportunity. Several researchers also adopted map-matching algorithms to improve indoor positioning accuracy. Ren and Guo [44] employed a 2D map-matching algorithm using CRF based on inertial data which improved positioning accuracy.

Geng and Xia [14] proposed a robust adaptive cubature Kalman filter algorithm for heading estimation. The heading and step length of each step was optimized by a Kalman filter to decrease positioning error. A calculation strategy for the heading angle of the 16-wind rose map based on the indoor map vector information was proposed, which improved pedestrian positioning accuracy and decreased the accumulation error. The robust adaptive Kalman filter algorithm was also used to fuse differential barometric altimetry and step frequency detection methods to estimate the optimum altitude. To improve the accuracy of positioning, Park and Lee [45] integrated the Integration approach (IA) and Parametric approach (PA) in PDR systems. When the direction of the person’s movement differed from the direction of the phone, they used PCA to estimate the direction and PA to estimate the step length. Wu and Ma [1] exploited human activity recognition and PDR components’ parameter adjustment according to each recognition activity. They defined two types of human activities: (a) steady-heading, i.e., ascending/descending stairs, stationary, normal walking, stationary stepping and lateral walking, and (b) non-steady-heading activities, i.e., door opening and turning. They employed SVM and DTree machine learning algorithms to recognize steady-heading activities. They also used an auto encoder-based deep neural network and a heading range-based method to detect non-steady-heading activities. The overall classification accuracy of their method was 98.44% and its average positioning error in a multi-story building was 1.79 m. However, their system was developed and tested by only two people and they considered only texting as phone-carrying mode.

The reviewed studies attempted to adjust the methods of step detection, step length estimation and heading determination based on motion states to improve the accuracy of the PDR. Few investigations, however, have adjusted the PDR components based on movement habits, walking speeds and user characteristics. Because the user moves at different speeds along a complex indoor path, using the PDR method without adjusting the parameters of its components based on various walking speeds causes errors in positioning. Moreover, according to the literature, detecting steps and estimating step length without considering users’ characteristics including gender, height and age led to significant positioning errors. In this paper, we improve the accuracy and robustness of the proposed method by adjusting PDR component parameters based on walking speeds, motion states and pedestrian characteristics, including gender and height.

## 3. The Proposed Method

**Figure 1:**
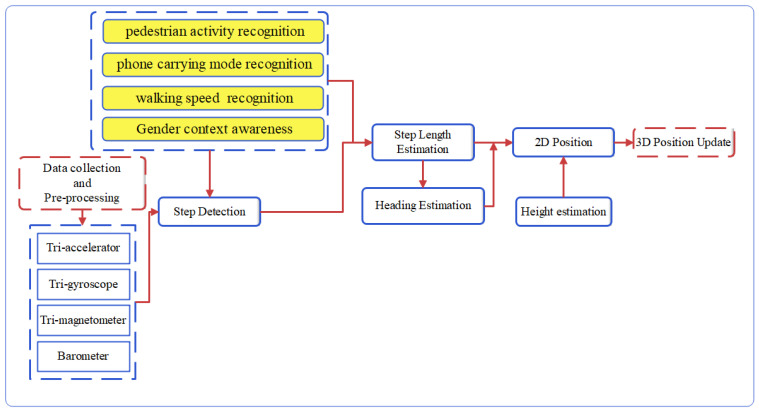
The structure of the proposed PDR system.

The proposed PDR system (Figure 1) includes data collection, data calibration, motion detection, step detection, step length estimation, heading determination and height estimation. Initially, the data are collected by volunteers of different heights and ages as they walked at different speeds and modes. Several errors occurred in the measurement data; thus, calibration was required and the appropriate features were extracted from the calibrated data and used in the classification algorithm. Different motion modes were distinguished with the combination of DTree and SVM algorithms. DTree was used for recognizing different phone-carrying modes, while the SVM was employed to detect different pedestrian activities. The parameters of step detection and step length estimation were adjusted based on gender, walking speed, pedestrian activity and phone-carrying mode. A gradient descent algorithm (GDA) was utilized to estimate the heading from the accelerometer, gyroscope and magnetometer data. Finally, movement altitude was estimated with pressure data and the 3D position was calculated by using the PDR equation.

### 3.1. PDR

According to (1), the PDR approach calculates the user’s location at each step based on his/her length, direction and location in the previous step [46].

(1) $$X_{i}=X_{i-1}+L_{i}\times \cos\Psi_{i}\;.\;Y_{i}=Y_{i-1}+L_{i}\times \sin\Psi_{i}$$

where $X_{i}$ and $Y_{i}$ represent the user’s estimated position in Step *i* and $L_{i}$ and $\Psi_{i}$ are the length and direction of the user’s movement in Step *i*, respectively. The initial location is assumed to be known and can be determined by default or by QR codes in the building.

### 3.2. Components of the PDR Positioning System

Positioning using the PDR method includes various components, such as movement state recognition, step detection, step length estimation and movement altitude estimation. Each of these components will be discussed as follows. 

#### 3.2.1. Preprocessing

**Figure 2:**
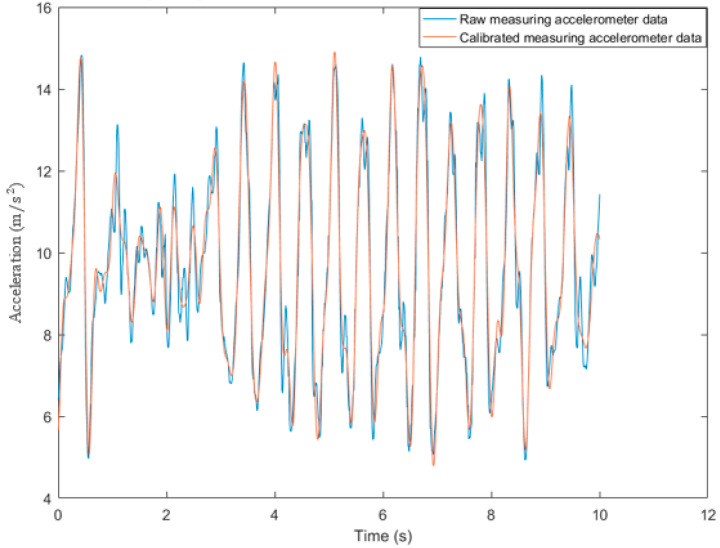
A low-pass filter for the acceleration signals with a cut-off frequency of 5 Hz to remove high-frequency noise.

Since smartphone-embedded inertial sensors are not very accurate, the raw sensor data are noisy. Before step detection and motion state recognition, a preprocessing process must be applied to eliminate the noise and errors. The low-pass filter, which uses a cut-off frequency of 5 Hz, softens and eliminates some high-frequency noises from the acceleration signals, allowing more accurate detection of pedestrian movements and reducing false step detection. In Figure 2, acceleration data are filtered using a low-pass filter with a cut-off frequency of 5 Hz.

**Figure 3:**
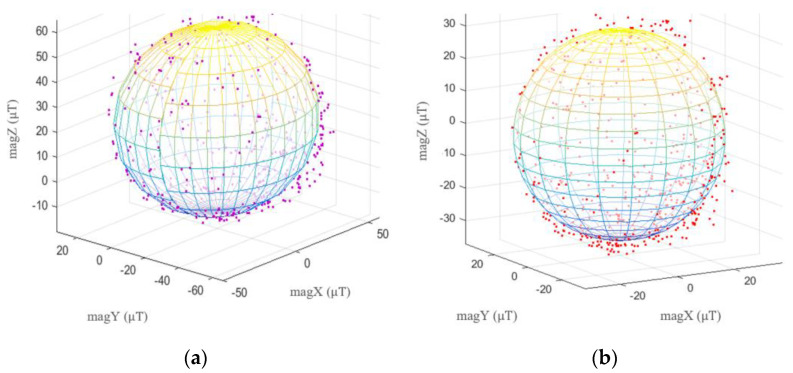
Magnetometer calibration; (**a**) Ellipsoid fitting model of raw magnetometer data; (**b**) Ellipsoid fitting model of calibration magnetometer data.

Smartphone magnetometers are easily affected by the magnetic fields of the local environment. There are two kinds of disturbances in the magnetometer data, hard iron disturbance and soft iron disturbance. Permanent magnet materials cause a hard iron disturbance, which affects the magnetometer’s values, similar to constant bias. Unlike hard-iron disturbances, soft-iron disturbances are caused by materials that influence or disturb but do not generate magnetic fields [47]. The magnetometer signals were calibrated using the least squares fitting ellipsoid method [48]. Ellipsoid fitting models from raw and calibrated magnetometer data are displayed in Figure 3a and Figure 3b, respectively.

#### 3.2.2. Classification of Different Motion States

**Figure 4:**
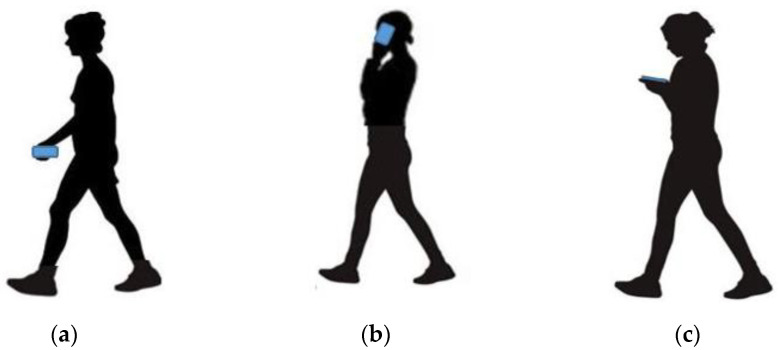
Different phone-carrying modes; (**a**) Swinging; (**b**) Calling; (**c**) Texting.

**Figure 5:**
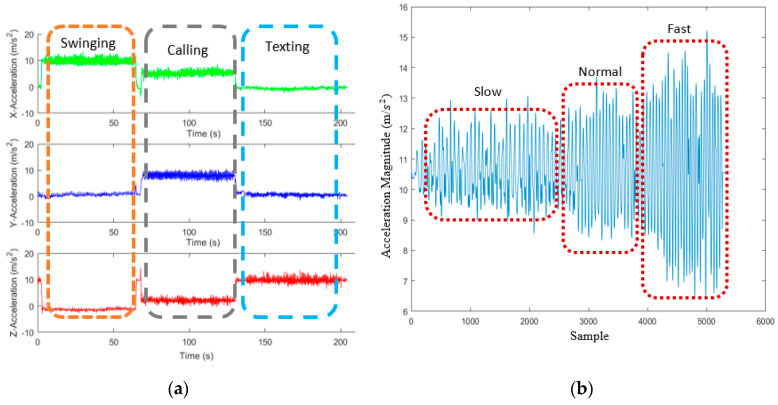
Comparison of acceleration data in different; (**a**) phone-carrying modes; (**b**) walking speeds.

Various activities were considered in the proposed method to enhance the robustness of PDR positioning, such as walking, ascending and descending stairs. Further, walking was classified as fast, normal, or slow, based on speed. Moreover, three phone-carrying modes, texting, calling and swinging, were considered. Based on Figure 4, when using a smartphone in texting mode, users hold it horizontally in front of their bodies and in calling mode they hold it vertically near their ears. In the swinging mode, users hold the smartphone in their hands and swing it. This study analyzed each movement state separately for women and men. Figure 5 compares the acceleration data values in different phone-carrying modes and walking speeds. Based on Figure 5b, as the speed increased, the range of acceleration during the steps rose. To recognize different motion states, three sensors, an accelerometer, gyroscope and barometer, were employed. The barometer had the lowest sampling rate, i.e., 10 Hz, whereas the sampling rates of the other two sensors were 100 Hz.

**Figure 6:**
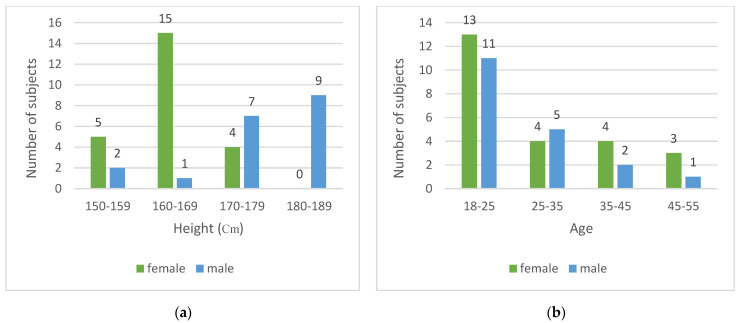
The experimenters’ characteristics; (**a**) Height range; (**b**) Age range.

To recognize different motion states, 15 features were extracted from the accelerometer, gyroscope and barometer data. The features were the average (except for the barometer), standard deviation (STD), the difference between the maximum and the minimum, skewness and zero crossing rate. To evaluate the performance of the classification, 43 experimenters of different ages and heights participated in the data collection, including 24 women and 19 men. The mean age and height of the men were, respectively, 27.5 ± 5.1 years and 171.1 ± 5.5 cm, while those of the women were, respectively, 27.54 ± 5.6 years and 159.6 ± 6.7 cm. More details about the experimenters’ height and age are shown in Figure 6.

The combination of 15 motion states including three phone-carrying modes (texting, calling, swinging) and five pedestrian activities (descending stairs, ascending stairs, fast walking, normal walking and slow walking) were considered as different motion states and the experimenters collected data in each state. After data collection, data segmentation was performed using a two-second sliding window with a 50% overlap and the sensor sampling frequency was 100 Hz. Each two-second sliding window is called an instance. Women and men had 6112 and 5336 instances in total, respectively. The instance number of each class is reported in Table 1.

**Figure 7:**
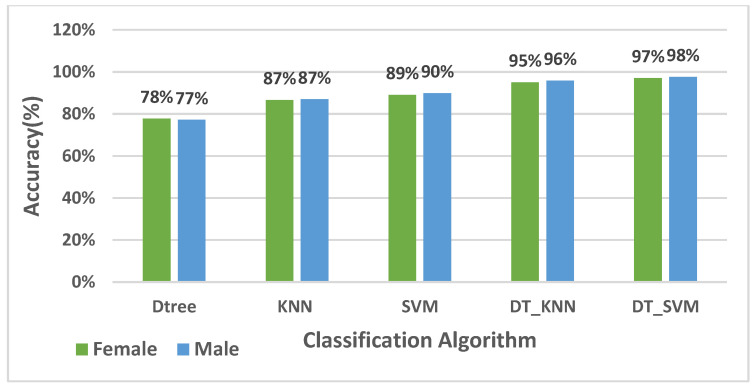
Comparison of the performance recognition of different algorithms based on the 5-fold cross-validation.

A combination of DTree and SVM algorithms was adopted to detect the motion states, including different phone-carrying modes and pedestrian activities. Based on 5-fold cross-validation [49], Figure 7 illustrates the recognition performance of women and men for DTree, KNN, SVM, a combination of DTree and SVM and a combination of DTree and KNN algorithms. According to Figure 7, a combination of DTree and SVM algorithms outperformed the mentioned algorithms in women and men with 97% and 98% accuracy, respectively.

**Figure 8:**
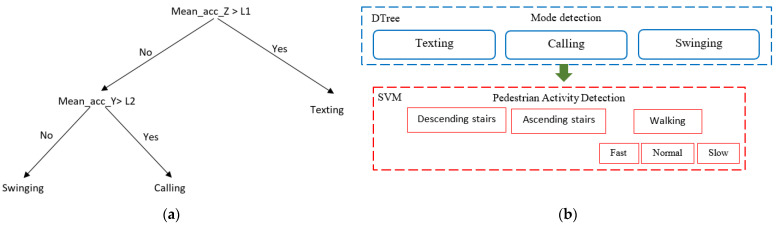
(**a**) DTree Model; (**b**) The combination of DTree and SVM to recognize motion states.

Using DTree and SVM algorithms, 15 combinations of phone-carrying modes and pedestrian activities were recognized. As shown in Figure 8b, DTree was used to detect phone-carrying modes, while SVM was utilized to detect pedestrian activities based on the identified phone-carrying mode. A DTree is a supervised learning algorithm and non-parametric classifier in the form of a tree and is composed of nodes, branches and leaves that are predicted classes. To predict class labels, DTree uses training data to infer decision rules [50]. DTree was used to detect the phone-carrying modes in this paper, where the average acceleration values of the X, Y and Z axes were used as the inputs. Accordingly, three rules were designed based on training data. The tree view of this DTree is presented in Figure 8a. L1 and L2 are the parameters of the DTree model and are estimated according to the pedestrian’s gender. L1 and L2 are (0.95,0.22) and (0.82,0.055) for females and males, respectively. According to the output of DTree, the SVM algorithm is used to determine pedestrian activities and walking speed. SVM is a supervised machine learning algorithm used for both classification and regression. The objective of this algorithm is to identify hyperplanes to separate data points into different classes, which is improved by mapping input feature data into a higher-dimensional feature space [51]. Accordingly, Kernel functions are utilized to map input feature data from a lower-dimensional space into a higher-dimensional space [52]. The radial basis function kernel is selected as the kernel function in this article. Input vectors of the SVM include average (except for barometer), STD, the difference between the maximum and minimum values, skewness and zero crossing rate of different sensors’ data, while the output is the motion state.

The recognition accuracy of the DTree algorithm based on 5-fold cross-validation is shown in Table 2. The average recognition accuracy of the phone-carrying modes was 98.7% for women and 99.7% for men. DTree confusion matrices for both men and women are given in Table 3, with the rows representing actual phone-carrying modes and the columns showing the detected phone-carrying modes. According to Table 3, texting and swinging modes were recognized correctly in both genders. Nevertheless, 2.9% and 0.9% instances of the calling mode were misrecognized as the swinging mode in women and men, respectively.

The recognition performance of SVM based on 5-fold cross-validation is given in Table 4. According to Table 4, the average recognition accuracy of pedestrian activities for different states was 95.5%. The SVM confusion matrices for both men and women are shown in Table 5, with the rows representing actual pedestrian activities and the columns showing the detected pedestrian activities. According to Table 5, in all six states, descending and ascending stairs were distinguished with over 97.9% accuracy. In addition, three types of walking speed were distinguished for women in texting, calling and swinging modes with an average accuracy of 94.2%, 94.6% and 93.2%, respectively. For men in texting, calling and swinging modes, three types of walking speeds were distinguished with an average accuracy of 92%, 94.4% and 95.9%, respectively.

#### 3.2.3. Step Detection

**Figure 9:**
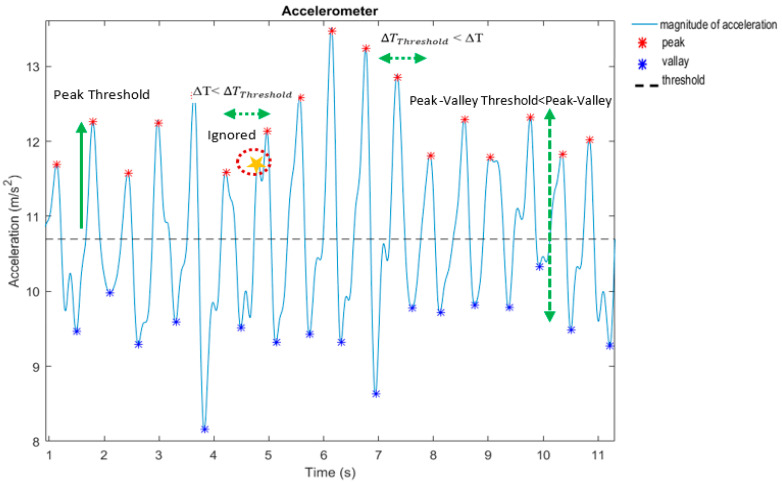
Thresholds of the peak detection method.

The peak detection method is used for step detection and its process is initiated by detecting the peak points in the accelerometer data. If the peak points are below the peak threshold or closer to the corresponding valley points than the peak valley threshold, they are eliminated. Additionally, peak points are removed if the time between them and the next peak point is less than the time threshold to prevent overcounting [20]. The mentioned thresholds are illustrated in Figure 9.

Based on pedestrian activity and walking speed, threshold values were determined empirically to improve the accuracy of step detection. Table 6 lists the relevant threshold values for pedestrian activities and walking speeds.

#### 3.2.4. Step Length Estimation

**Figure 10:**
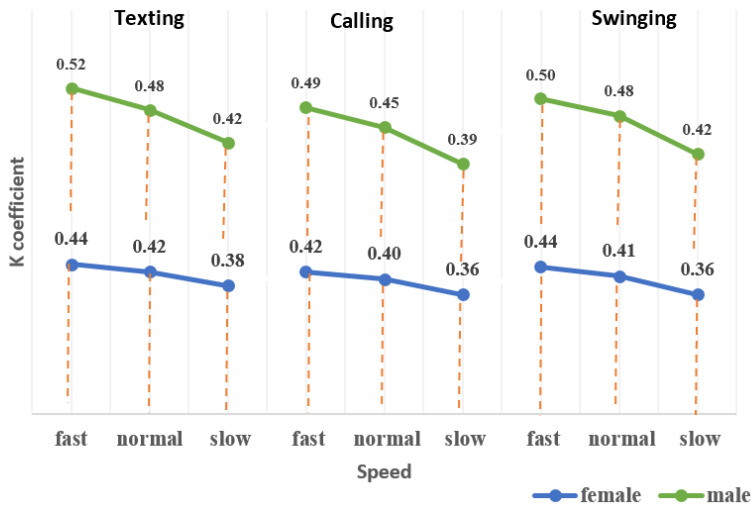
Estimated k values.

Weinberg’s model, which uses the quartic root of the difference between the maximum and minimum of *z*-axis acceleration, was adopted to estimate step length [26]. Forty people, including 20 men and 20 women of different ages and heights, walked along a 20-m path at different speeds while carrying their smartphones in texting, calling and swinging modes. Then, the K coefficient was estimated for each state using the least squares technique. The mathematical equation of Weinberg’s model is presented in (2).

(2) $$S=k\;\times \;\sqrt[{4}]{a_{max}-a_{min}\;}$$

where $a_{max}$ is the maximum acceleration in each step, $a_{min}$ denotes the minimum acceleration in each step and k represents the coefficient estimated based on different walking speeds, genders and phone-carrying modes. According to Figure 10, the k coefficient for men was higher than for women at the same speed in each motion mode. Furthermore, the k value increased for higher walking speeds.

**Figure 11:**
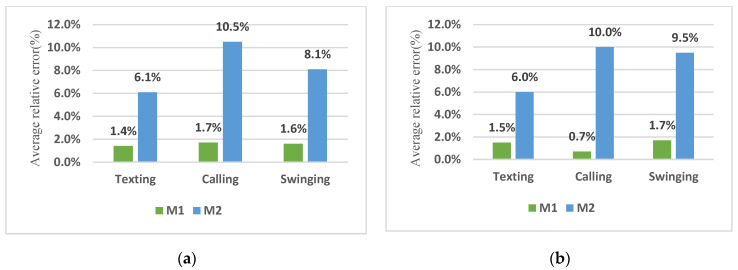
Comparison of the average relative error values of distance estimation; (**a**) Straight path; (**b**) Rectangular path.

The step length was calculated using the Weinberg model. To assess the performance of the step length estimation, four experimenters of different ages and heights participated, including two women and two men. The mean age and height of the men were respectively 25.5 ± 6 years and 175.1 ± 6.5 cm, while those of the women were respectively 25.5 ± 5.5 years and 161.5 ± 7.7 cm. In this section, gender and walking speeds are called effective parameters. Assuming that pedestrian activity is detected, for walking, the k values were selected based on effective parameters and step length was estimated based on the selected k; otherwise, for ascending and descending stairs, the step length was assumed 0.3 m regardless of the effective parameters. Table 7 and Table 8 present the estimated distance for two paths, including a straight path of 56.7 m and a rectangular path of 79.9 m, respectively. These tables also include comparisons of distance error values by considering and neglecting effective parameters. Column M2 in Table 7 and Table 8 represents the results of the step length estimation without considering the effective parameters. According to Table 7, the relative distance errors of the straight path for texting, calling and swinging modes were 1.4% and 6.1%, 1.7% and 10.5% and 1.6% and 8.1%, respectively, when considering and neglecting the effective parameters. As shown in Table 8, the relative distance errors of the rectangular path for texting, calling and swinging modes were respectively 1.5% and 6%, 0.7% and 10% and 1.7% and 9.5% when considering and neglecting effective parameters. Based on Figure 11a,b, the distance estimation accuracy was improved significantly in both straight and rectangular paths by considering the effective parameters.

**Figure 12:**
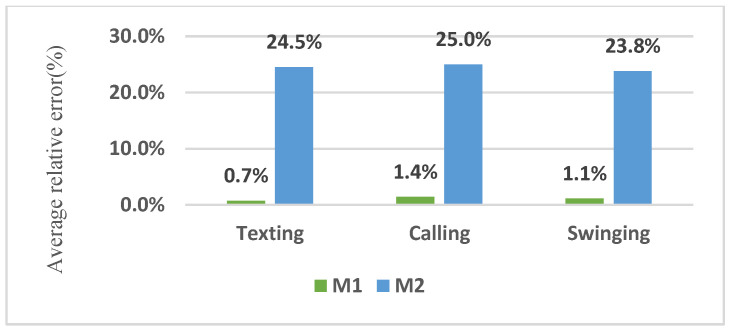
Comparison of the average relative errors of distance estimation in the 3D trajectory.

According to Table 9, the relative distance errors of 3D paths for texting, calling and swinging modes were 0.7% and 24.5%, 1.4% and 25% and 1.1% and 23.8%, respectively, when considering and neglecting the effective parameters and activity detection. According to Figure 12, in addition to considering the effective parameters, recognizing the ascending or descending stairs significantly reduced the distance estimation error in the 3D trajectory.

#### 3.2.5. Heading Estimation

The GDA algorithm (Algorithm 1) was used for heading estimation. The mathematical analysis of the gradient descent algorithm for heading estimation has been widely covered in the literature [32,53]; therefore, only a summary of the equations used for heading estimation is presented and discussed in this section. This algorithm uses accelerometer and magnetometer signals to calculate the gyroscope measurement error as quaternion derivatives. System inputs include acceleration, gyroscopes and magnetometer sensors. Generally, GDA assumes that magnetic data within a building are subject to external magnetic disturbances. To increase accuracy, magnetic data with a wide fluctuation range relative to the reference magnetic field is avoided [53]. In this algorithm, stability is the result of magnetic field stability. $mag^{s}$, $mag^{Earth}$ and mag_stability_threshold represent the measured magnetometer field, the Earth’s local reference magnetic field and the threshold to limit the magnetic field interference, respectively. Vector F represents the error vector between the estimated value and the measured data of acceleration and magnetometer. If magnetic stability = 1, the magnetometer data are used; otherwise, gyroscope and accelerometer data are combined. Consequently, in the case of stable magnetic information, error vector F(${\hat{q}}_{t-1}\;.\;mag$) and, otherwise, error vector F(${\hat{q}}_{t-1}\;.\;\mathrm{acc}$) is used to correct the ${\dot{q}}_{t}$ and the final optimal quaternion value ${\hat{q}}_{t}$ is obtained. This algorithm requires a parameter called β, which represents the measurement error of the gyroscope [32]. β was assumed to be 0.05 in this paper.
**Algorithm 1.** GDA algorithm**Input**: acc → measured acceleration, ω → measured angular velocity, mag → measured magnetometer,$\;G_{acc}$ → earth’s gravity, $G_{mag}$ → magnetic field vectors**Output**: ${\hat{q}}_{t}$→ updated quaternions 1.$S_{\omega }=\left[0{\;\omega }_{x}{\;\omega }_{y}{\;\omega }_{z}\right]$2.  If ( | ||${\mathrm{mag}}^{s}$|| -${\mathrm{mag}}^{\mathrm{Earth}}$ | < mag_ stability_thereshold ) then3.   stability = 14.else then5.     stability = 06.end if7.F(${\hat{q}}_{t-1}\;.\;\mathrm{acc}$) = $({\hat{q}}_{t-1}^{*}⊗$ $G_{\mathrm{acc}})\;⊗{\hat{q}}_{t-1}$- acc8.If (stability) then9. F(${\hat{q}}_{t-1}\;.\;\mathrm{mag}$) = ${\hat{q}}_{t-1}^{*}⊗$ $G_{\mathrm{mag}}\;⊗{\hat{q}}_{t-1}$ − mag
10. ∇ F(q) = ${\left[\begin{array}{c} J\left({\hat{q}}_{t-1\;}.{\mathrm{acc}}_{t}\right)\\ J\left({\hat{q}}_{t-1}\;.\mathrm{mag}\right) \end{array}\right]}^{T}\left[\begin{array}{c} F\left({\hat{q}}_{t-1}\;.\;\;{\mathrm{acc}}_{t}\right)\\ F\left({\hat{q}}_{t-1}\;.\;\mathrm{mag}\right) \end{array}\right]$11.else then12. $\nabla \;F\left(q\right)=J^{T}\left({\hat{q}}_{t-1\;}.{\mathrm{acc}}_{t}\;\right)F({\hat{q}}_{t-1}\;.{\;\mathrm{acc}}_{t}$)13.end if14.${\dot{q}}_{t}=\frac{1}{2}{\hat{q}}_{t-1\;}⊗S_{\omega }-\beta \frac{\nabla \;F\left(q\right)}{\left|\nabla \;F\left(q\right)\right|}$15.$q_{t}={\hat{q}}_{t-1}+{\dot{q}}_{t}\;\Delta t$16.${\hat{q}}_{t}$ = $\frac{q_{t}}{\left|q_{t}\right|}$17.Return ${\hat{q}}_{t}$


According to (3), the heading was estimated based on Eulerian angles [54]. In (3), the scalar part of *q* is *q*_0_ and its vector part is (*q*_1_, *q*_2_, *q*_3_).

(3) $$yaw=\tan^{-1}\left(\frac{2\left(q_{2}q_{3}-q_{0}q_{1}\right)}{q_{0}^{2}-q_{1}^{2}+q_{2}^{2}-q_{3}^{2}}\right)$$

### 3.3. Calculation of the Pedestrians’ Movement Height

**Figure 13:**
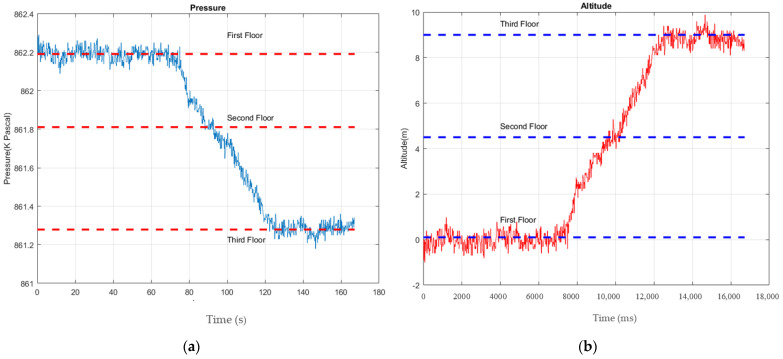
(**a**) The sensor’s measurements; (**b**) altitude values derived from pressure sensor measurements.

According to (4), pressure sensor measurements were used for height estimation [55].

(4) $$h_{t}=h_{0}+\frac{R\times T_{0}\times \ln\left(\frac{P_{t}}{P_{0}}\right)}{-gM}$$

where *h*_0_ is the initial height in meters, *R* is the universal gas constant equal to 8.31432$\;\frac{\mathrm{NM}}{\mathrm{molk}}$, *T*_0_ is the temperature in Kelvin and *P*_0_ and *P_t_* are, respectively, the initial atmospheric pressure and the atmospheric pressure in Pascal at time *t*. Moreover, *g* is the magnitude of local gravity acceleration equal to 9.806 $\frac{m}{s^{2}}\;$ and *M* is the average molar mass of air equal to 0.0289644$\frac{\mathrm{Kg}}{\mathrm{mol}}$. Figure 13a demonstrates the pressure sensor measurements. Building floors are distinguished based on altitudes derived from pressure sensor measurements. As shown in Figure 13b, walking initiates on the first level. As the pedestrian walks upstairs, his/her height increases from zero on the first level to 9 m on the third.

**Figure 14:**
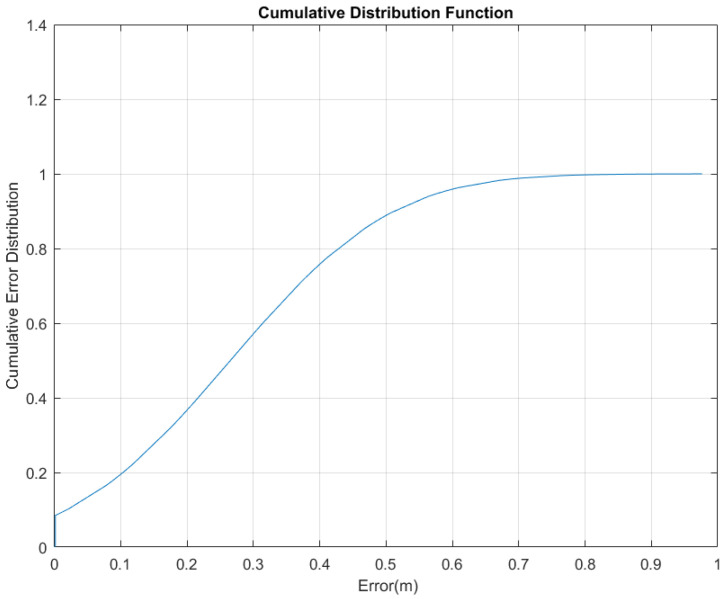
The CDF of the height estimation error.

The values of altitude estimation error were calculated based on differences between the estimated and the actual altitude values. The mean and standard divisions of altitude estimation error were 0.26 m and 0.19 m, respectively. The cumulative disturbance function (CDF) was used to further analyze the absolute estimation altitude errors. According to Figure 14, the 80% probability of altitude estimation error is less than 0.42 m. This value cannot affect floor detection in this building because the floors’ level elevation differs by 4.5 m and it is comparatively small.

## 4. Positioning Experiments and Assessment

**Figure 15:**
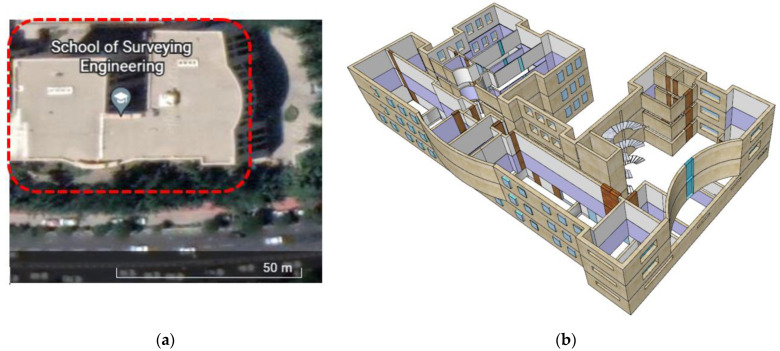
The case study area; (**a**) the area on the google map; (**b**) The interior environment of the area.

The experiments were conducted in the building of the School of Surveying and Geospatial Engineering at the University of Tehran (Iran). As shown in Figure 15, it is a normal multi-story building with classrooms and offices. Using the multi-story building plan in Google SketchUp software, a three-dimensional model was generated for a better representation of the studied area. This section of the paper contains the presentation of the results followed by the analysis and discussion of the results.

**Figure 16:**
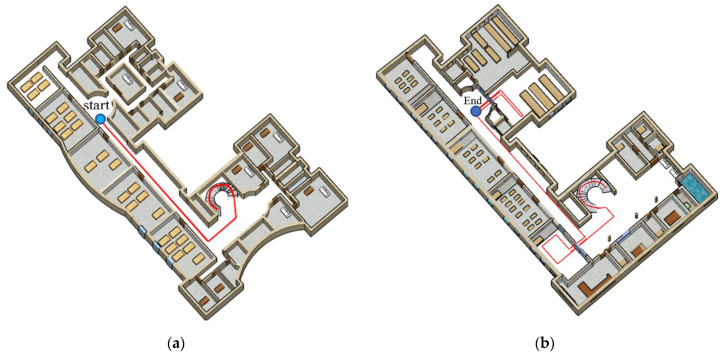
Designed path; (**a**) Third floor; (**b**) First floor.

To evaluate the performance of the positioning, experimenters of different ages and heights, two women and two men, participated. The average age and height of the men were, respectively, 28 years and 174.1 cm and, respectively, 25 years and 164 cm for women. The experimenters moved along the designed path of 159.2 m using four smartphones (Samsung Galaxy S4, Xiaomi Poco F2 Pro, Samsung Galaxy Note 10 and iPhone 13 Pro). As shown in Figure 16, the starting point of the test was on the third floor and its ending point was on the first floor.

In this experiment, reference points were set at the ending and starting points of staircases and turning points, which are significant landmarks for estimating error positioning along the designed path. In this regard, we asked the experimenters to step over the reference point as precisely as possible. The positioning error is estimated by calculating the Euclidean distance between the estimated and actual coordinates of reference points. Furthermore, the estimated distance is the total length of the step lengths and the distance estimation error is the difference between the estimated distance and the actual distance. In this section, positioning with the PDR method based on the recognition of walking, ascending and descending stairs is called activity-based PDR. However, the proposed method recognizes walking speed in addition to activities. It also considers different genders in each activity and the walking speed.

### 4.1. Texting Mode Positioning Experimentation

According to Table 10, for women, the average relative distance error decreased from 21.38% to 4.97% and 0.88% using an activity-based PDR and the proposed method, respectively. For men, by using the activity-based PDR and the proposed method, the relative distance error decreased from 21.02% to 8.12% and 1.15%, respectively. The average and STD of positioning error values are presented in Table 10. For women, the typical PDR method had higher error values compared to the other two methods. It had an average error value of 3.02 m and an STD of 2.82 m. The mean and standard errors of the activity-based PDR were 2.52 m and 1.71 m, respectively, confirming that it is more accurate than the typical PDR. The proposed method had the lowest error values among the three methods and its average and STD were 1.28 m and 0.76 m, respectively, confirming that it is more accurate than the other two methods. For men (Table 10), the typical PDR method had higher error values than the other two methods. It had an average error value of 4.07 m and an STD of 2.09 m. The activity-based PDR’s mean and STD errors were 3.33 m and 1.61 m, respectively. Moreover, the proposed method had the lowest error values and its average and STD were 1.26 m and 0.68 m, respectively, demonstrating that it performs best among the three methods. Based on Table 10, the PDR algorithm performance was improved by activity, walking speed and gender detection. By detecting activities, the average positioning error was reduced by 0.5 m for women and 0.74 m for men. Walking speed detection and gender were also important factors for improving PDR performance in addition to activity detection. The proposed method decreased the average errors by 1.24 m for women and 2.07 m for men compared to the recognition of activity alone.

**Figure 17:**
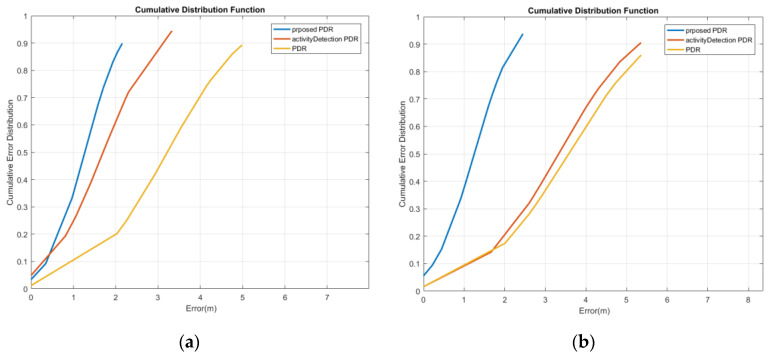
Cumulative error distribution of the texting mode; (**a**) woman; (**b**) man.

The cumulative error distribution curve of reference point estimation for the first woman is illustrated in Figure 17a. This curve indicates that the mean position error with a probability of 95% has decreased from 4.85 m to 3.31 m and 2.09 m using the activity-based PDR and the proposed method, respectively. Moreover, as shown in Figure 17b, for the male subject 1, by using the activity-based PDR and the proposed method, the mean position error with a probability of 95% decreased from 5.33 m to 5.06 m and 2.17 m, respectively.

**Figure 18:**
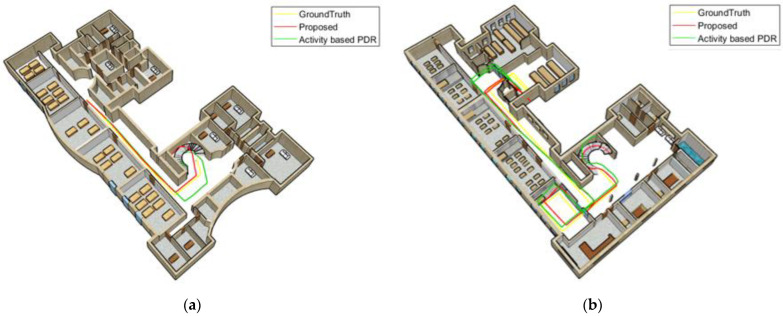
Texting woman; (**a**) third floor; (**b**) first floor.

**Figure 19:**
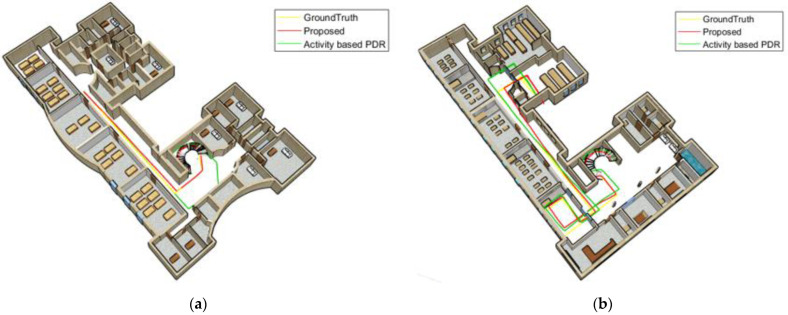
Texting man; (**a**) the third floor; (**b**) the first floor.

Figure 18 and Figure 19 compare the position results of the two strategies, including activity-based PDR and the proposed method, from a 3D perspective for the Female and Male subject 1. Figure 18a and Figure 19a show the positioning results for the third floor, while Figure 18b and Figure 19b depict the positioning results for the first floor. The yellow line shows the ground truth, the green line represents the results of the activity-based PDR and the red line denotes the results of the proposed method. As shown in these figures, due to neglecting speed changes, the green line drifts away from the reference line at some points. Based on the proposed method (red line), the position is determined much more accurately and its distance from the reference path is significantly reduced since the step length error is decreasing when considering walking speed variations and gender differences. 

### 4.2. Calling Mode Positioning Experimentation

According to Table 11, for women, the average of relative distance errors decreased from 16.3% to 4.21% and 0.67% using the activity-based PDR and the proposed method, respectively. For men, by using the activity-based PDR and the proposed method, the relative distance error decreased from 12.84% to 4.11% and 0.93%, respectively. The average and STD of positioning error values are presented in Table 11. For women, the typical PDR method had higher error values compared to the other two methods. It had an average error value of 3.52 m and an STD of 1.76 m. The activity-based PDR had a mean error of 1.78 m and an STD of 0.94 m, confirming its higher accuracy compared to typical PDRs. As compared to the other two methods, the proposed method had the lowest error values, with an average and STD of 0.98 m and 0.51 m, respectively. For men (Table 11), the typical PDR method had higher error values than the other two methods. It had an average error value of 4.23 m and an STD of 2.31 m. Besides, the activity-based PDR’s mean and STD errors were 2.49 m and 1.12 m, respectively. Furthermore, the proposed method had the lowest error values and its average and STD were 1.17 m and 0.7 m, respectively, indicating that it performs the best out of the three. Results in Table 11 indicate that the performance of the PDR algorithm is improved by activity, walking speed and gender detection. By detecting activities, the average positioning error was reduced by 1.74 m for women and 1.73 m for men. In addition to activity detection, PDR performance was enhanced by detecting walking speed and gender. The proposed method decreased the average errors by 0.8 m for women and 1.32 m for men compared to the recognition of activity alone.

**Figure 20:**
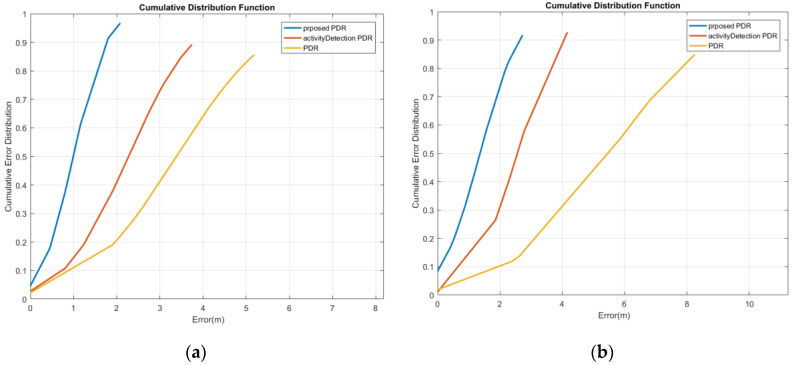
Cumulative error distribution of the calling mode; (**a**) woman; (**b**) man.

The cumulative error distribution curve of reference point estimation for the first woman is illustrated in Figure 20a. This curve indicates that the mean position error with a probability of 95% decreased from 5.14 m to 3.59 m and 1.92 m using the activity-based PDR and the proposed method, respectively. Moreover, as shown in Figure 20b, for the first man, by using the activity-based PDR and the proposed method, the mean position error with a probability of 95% decreased from 8.24 m to 4.16 m and 2.72 m, respectively.

**Figure 21:**
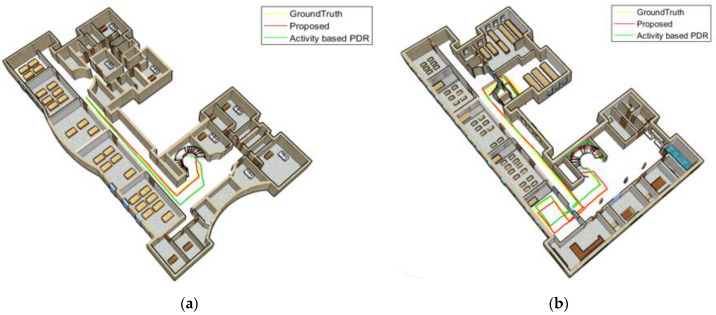
A calling woman; (**a**) third floor; (**b**) first floor.

**Figure 22:**
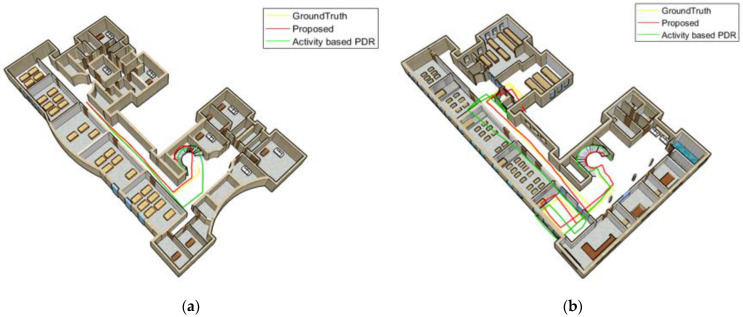
A calling man; (**a**) third floor; (**b**) first floor.

Figure 21 and Figure 22 compare the position results of the two strategies, including activity-based PDR and the proposed method, from a 3D perspective for the first woman and man. Figure 21a and Figure 22a illustrate the positioning results for the third floor, whereas Figure 21b and Figure 22b depict the positioning results for the first floor. The yellow line represents the ground truth. According to these figures, the green line associated with activity-based PDR drifts away from the reference line at some points. Because gender and walking speed are considered in the proposed method (red line), step length errors and distance from the reference path significantly declined.

### 4.3. Swinging Mode Positioning Experimentation

According to Table 12, for women, the average relative distance error decreased from 17.39% to 5.76% and 0.93% using the activity-based PDR and the proposed method, respectively. For men, by using the activity-based PDR and the proposed method, the average relative distance error decreased from 11.93% to 3.71% and 0.77%, respectively. For women, according to Table 12, the typical PDR method had higher error values compared to the other two methods. It had an average error value of 3.81 m and an STD of 1.97 m. Compared to the typical PDR, the activity-based PDR had a mean error of 2.15 m and an STD of 1.25 m, confirming its higher accuracy. In comparison to the other two methods, the proposed method had the lowest error values, with an average and STD of 1.29 m and 0.64 m, respectively. For men (Table 12), the typical PDR method had higher error values than the other two methods. It had an average error value of 5.87 m and an STD of 3.53 m. Besides, the activity-based PDR’s mean and STD errors were 2.52 m and 1.37 m, respectively. Furthermore, the proposed method had the lowest error values and its average and STD were 1.25 m and 0.66 m, respectively, demonstrating that it performs the best out of the three. Results in Table 12 indicate that the performance of the PDR algorithm is improved by activity, walking speed and gender detection. By detecting activities, the average positioning error was reduced by 1.66 m for women and 3.35 m for men. In addition to activity detection, the PDR performance was promoted by detecting walking speed and gender. The proposed method reduced the average errors by 0.86 m for women and 1.27 m for men compared to the recognition of activity alone.

**Figure 23:**
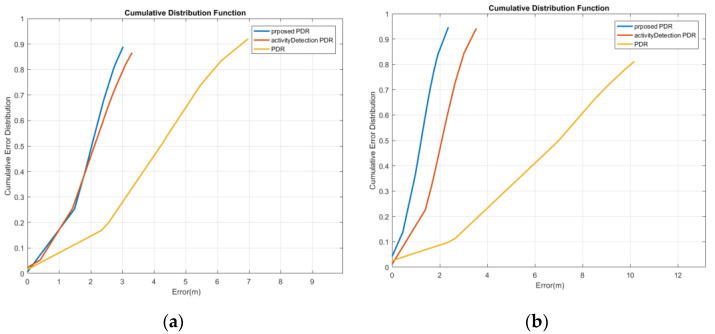
Cumulative error distribution of the swinging mode; (**a**) woman; (**b**) man.

The cumulative error distribution curve of reference point estimation for the first woman is illustrated in Figure 23a. It demonstrates that the mean position error with a probability of 95% decreased from 6.49 m to 3.19 m and 2.88 m using the activity-based PDR and the proposed method, respectively. Furthermore, as depicted in Figure 23b, for the first man, by using the activity-based PDR and the proposed method, the mean position error with a probability of 95% decreased from 10.09 m to 3.2 m and 2.12 m, respectively.

**Figure 24:**
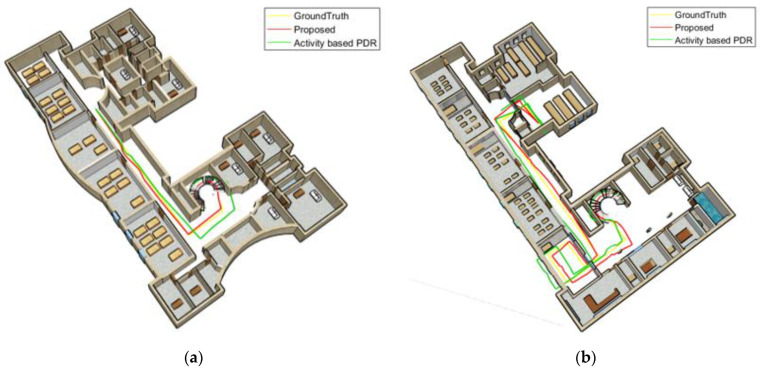
Swinging woman; (**a**) third floor; (**b**) first floor.

**Figure 25:**
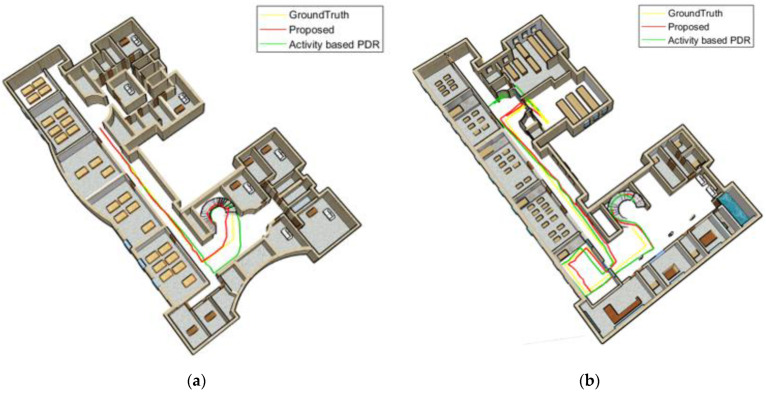
Swinging man; (**a**) third floor; (**b**) first floor.

Figure 24 and Figure 25 compare the position results of the two strategies, including the activity-based PDR and the proposed method, from a 3D perspective for the first woman and man. Figure 24a and Figure 25a display the positioning results for the third floor, while Figure 24b and Figure 25b show the positioning results for the first floor. The proposed method (red line) reduced step length errors and its distance from the reference path significantly declined. At some points, however, the green line (associated with the activity-based PDR) veered off the reference line. 

**Figure 26:**
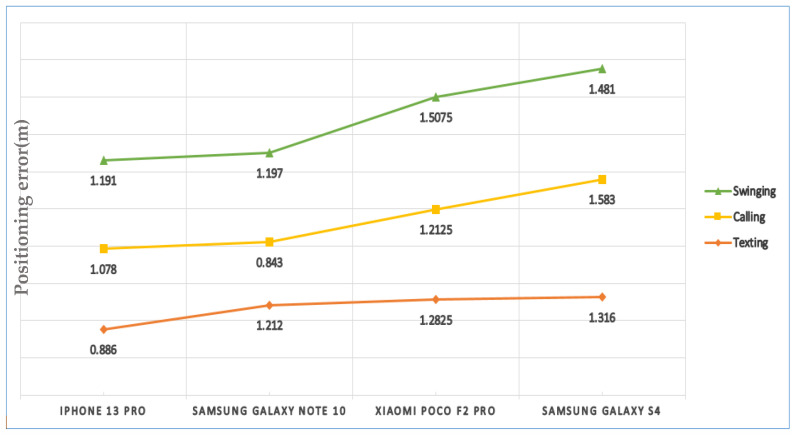
Comparison of the average positioning errors of different smartphone models.

Figure 26 compares the average positioning error of four smartphone models, including Samsung Galaxy S4, Xiaomi Poco F2 Pro, Samsung Galaxy Note 10 and iPhone 13 Pro for various phone-carrying modes. As compared to the other smartphone models, positioning with iPhone 13 Pro and Samsung Galaxy S4 had the lowest and highest error values, with an average of 1.05 m and 1.46 m, respectively.

According to Table 13, key parameters were not considered by Wang and Luo [17], Geng and Xia [14], Park and Lee [45], Wu and Ma [1] and Saadatzadeh and Ali Abbaspour [56]. The experiments by Wang and Luo [17] were carried out on a trajectory of 146 m for the texting mode and the distance error was 1.91 m. In Geng and Xia [14], the experiments were conducted on a trajectory of 118 m for texting and the positioning error was 1.61 m, whereas in Park and Lee [45] the experiments were performed on a trajectory of 58 m for texting and swinging and the positioning errors were 1.61 m and 3.41 m, respectively. A trajectory of 210 m was used by Wu and Ma [1] and, for the texting mode, the positioning error was 2.68 m. In Saadatzadeh and Ali Abbaspour [56], the experiment was conducted on a trajectory of 148.53 m for texting, calling and swinging modes, with distance errors of 2.68 m, 3.82 m and 8.39 m, respectively. In Klein and Solaz [16], the step length parameters were optimized based on walking speed, as shown in Table 13. The experiments were conducted on a trajectory of 21.4 m involving texting, calling and swinging modes, with distance errors of 0.38 m, 0.107 m and 0.47 m, respectively. In addition to the walking speed, Gu and Khoshelham [18] and Lu and Wu [43] also considered the pedestrian characteristics. As reported by Gu and Khoshelham [18], on a trajectory of 100 m, the distance error was 3.01 m. Furthermore, Lu and Wu [43] studied the texting mode on a trajectory of 100 m, with a distance error of 1.74 m. In this study, by optimizing the parameters of step detection and step length estimation based on different walking speeds, motion states and gender, the distance errors in texting, calling and swinging modes were 1.68 m, 1.27 m and 1.35 m, respectively, which improved significantly.

## 5. Conclusions

This study used adaptive PDR positioning. Different pedestrian activities, phone-carrying modes and walking speeds were detected using a combination of SVM and DTree algorithms to promote the robustness of PDR positioning. Additionally, each motion state was investigated separately based on the experimenter’s gender. The proposed classification approach recognized various motion states and walking speeds with a recognition accuracy of 95% for women and 97% for men. After motion state detection, motion states, walking speeds and gender were utilized to adjust the parameters of step counting and step length estimation methods separately for each motion state and gender. The goal was to enhance the robustness of PDR positioning. Using the optimization parameters, the absolute distance estimation error for texting, calling and swinging modes were respectively 1.4 m, 1.06 m, 1.48 m for women, 1.83 m, 1.48 m and 1.22 m for men on a trajectory of 159.2 m. The average absolute positioning error values of the proposed method for three phone-carrying modes, including texting, calling and swinging, were 1.28 m, 0.98 m and 1.29 m for women and 1.26 m, 1.17 m and 1.25 m for men in a multi-story building on a trajectory of 159.2 m. The proposed method promoted step length estimation and position accuracy with robust PDR method, by utilizing embedded smartphone sensors. Future studies can consider other smartphone-carrying modes such as pockets and bags and more pedestrian activities such as running and lateral walking.

## Figures and Tables

**Table 1:** The instance number of each class for the test.

Gender	Mode	Downstairs	Upstairs	Fast Walking	Normal Walking	Slow Walking
Female	Texting	572	332	259	223	759
	Calling	534	303	156	334	651
	Swinging	561	297	185	303	643
Male	Texting	513	352	238	299	537
	Calling	522	320	121	149	565
	Swinging	498	303	198	207	514

**Table 2:** The recognition accuracy of DTree algorithms based on the 5-fold cross-validation.

Gender	1	2	3	4	5	Average	STD
Female	99.1%	98.8%	98.0%	98.8%	98.8%	98.7%	0.004
Male	99.70%	99.50%	99.70%	99.50%	100%	99.7%	0.002
Average						99.2%	0.005

**Table 3:** Confusion Matrix of the DTree Algorithm.

Genders	Female			Male		
Modes	Texting	Calling	Swinging	Texting	Calling	Swinging
Texting	100%	0%	0%	100.0%	0%	0%
Calling	0%	97.1%	2.9%	0%	99.1%	0.9%
Swinging	0%	0%	100%	0%	0%	100%

**Table 4:** Recognition performance of SVM algorithms based on 5-fold cross-validation.

Mode	Gender	1	2	3	4	5	Average	STD
Texting	Female	96.2%	96.5%	96.5%	96.2%	96.1%	96.3%	0.002
	Male	92.7%	93.5%	93.7%	93.7%	94.6%	93.7%	0.006
Calling	Female	94.6%	94.8%	95.0%	95.2%	95.3%	95.0%	0.003
	Male	96.1%	96.9%	97.3%	96.7%	96.5%	96.7%	0.004
Swinging	Female	94.9%	94.8%	94.5%	94.5%	95.2%	94.8%	0.003
	male	96.4%	96.8%	96.5%	96.7%	96.7%	96.6%	0.002
Average							95.5%	0.011

**Table 5:** Confusion Matrix of the SVM Algorithm.

		Gender									
Modes		Female					Male				
	Activities	DS	US	FW	NW	SW	DS	US	FW	NW	SW
**Texting**	Downstairs	97.9%	0%	1.6%	0%	0.5%	99.5%	0%	0%	0.5%	0%
	Upstairs	0%	99.1%	0%	0%	0.9%	0%	100%	0%	0%	0%
	Fast walking	3.4%	0%	95.4%	1.1%	0%	1.3%	0%	91.1%	2.5%	5.1%
	Normal walking	0%	0%	2.7%	88.8%	8.5%	0%	0%	1%	93%	6.0%
	Slow walking	0%	0%	0%	1.6%	98.4%	0%	0%	2.8%	5.1%	92.1%
**Calling**	Downstairs	98.1%	0.5%	1.4%	0%	0%	98.2%	0%	0%	0%	1.8%
	Upstairs	0%	99%	0%	1%	0%	0.9%	99.1%	0%	0%	0%
	Fast walking	0%	0%	96.2%	3.8%	0%	0%	0%	95%	5%	0%
	Normal walking	0%	0%	0%	92.8%	7.2%	0%	0%	2%	89.8%	8.2%
	Slow walking	0%	0%	0%	5.1%	94.9%	0%	0%	1.1%	1.1%	97.9%
**Swinging**	Downstairs	97.9%	0%	0%	2.1%	0%	98%	1.0%	0%	1.0%	0%
	Upstairs	0%	98.5%	0%	0%	1.5%	0.9%	98.2%	0%	0%	0.9%
	Fast walking	0%	0%	95.2%	4.8%	0%	0%	0%	100%	0%	0%
	Normal walking	0%	0%	1%	90.1%	8.9%	0%	0%	1.4%	91.3%	7.2%
	Slow walking	0%	0%	0.9%	4.7%	94.4%	0%	0%	1.2%	2.3%	96.5%

**Table 6:** Threshold values.

Pedestrian Activity	Speed	Peak Threshold $\left(m∕s^{2}\right)$	Peak-Valley Threshold $\left(m∕s^{2}\right)$	Time Difference Threshold (s)
	Slow	11.2	1.5	0.5
	Normal	11.4	2	0.4
	Fast	11.6	2.5	0.3
**Ascending and descending stairs**	-	11.75	2.5	0.2

**Table 7:** Distance estimation of the straight path.

Mode	Gender	Subject	Steps Count			Steps Count Error (%)		Distance (m)			Absolute Distance Error (m)		Relative Distance Error (%)	
			Actual	M1*	M2*	M1*	M2*	Actual	M1*	M2*	M1*	M2*	M1*	M2*
Texting	Female	1	102	99	99	3.3%	3.4%	56.2	55.8	60.6	0.4	4.4	0.8%	7.8%
		2	100	99	99	1.4%	1.4%	56.2	55.7	60.3	0.5	4.1	1.0%	7.3%
	Male	1	72	70	69	3.4%	3.2%	56.2	55.5	51.3	0.7	4.9	1.2%	8.7%
		2	80	78	85	2.5%	5.3%	56.2	54.7	56.6	1.5	0.4	2.6%	0.6%
	Average					2.6%	3.3%	56.2	55.4	57.2	0.8	3.4	1.4%	6.1%
Calling	Female	1	102	104	103	2.0%	1.0%	56.2	57.2	64.4	1.0	8.2	1.7%	14.5%
		2	99	100	100	1.0%	1.0%	56.2	56.4	63.9	0.2	7.7	0.3%	13.7%
	Male	1	71	72	72	1.6%	1.6%	56.2	54.6	52.1	1.6	4.1	2.9%	7.4%
		2	79	82	89	3.8%	9.6%	56.2	55.2	59.7	1.0	3.5	1.7%	6.3%
	Average					2.1%	3.3%	56.2	55.8	60.0	0.9	5.9	1.7%	10.5%
Swinging	Female	1	99	102	102	3.0%	3.0%	56.2	57.1	61.8	0.9	5.6	1.6%	9.9%
		2	101	104	103	3.0%	2.0%	56.2	57.3	61.1	1.1	4.9	2.0%	8.7%
	Male	1	69	69	72	0.4%	3.2%	56.2	55.7	52.1	0.5	4.1	0.9%	7.4%
		2	76	78	84	2.6%	7.6%	56.2	55.1	52.7	1.1	3.5	1.9%	6.3%
	Average					2.2%	3.9%	56.2	56.3	56.9	0.9	4.5	1.6%	8.1%

M1*: the results of the step length estimation considering the effective parameters. M2*: the results of the step length estimation without considering the effective parameters.

**Table 8:** Distance estimation of the rectangular path.

Mode	Gender	Subject	Steps Count			Steps Count Error (%)		Distance (m)			Absolute Distance Error (m)		Relative Distance Error (%)	
			Actual	M1*	M2*	M1*	M2*	Actual	M1*	M2*	M1*	M2*	M1*	M2*
Texting	Female	1	130	133	132	2.6%	1.4%	79.9	80.2	85.2	0.3	5.3	0.3%	6.6%
		2	130	132	136	2.4%	6.4%	79.9	77.1	77.9	2.8	2.0	3.5%	2.5%
	Male	1	124	124	123	0.1%	1.0%	79.9	78.2	82.1	1.7	2.2	2.1%	2.8%
		2	99	101	101	2.1%	2.0%	79.9	80.1	70.3	0.2	9.6	0.2%	12%
	Average					1.8%	2.7%	79.9	78.9	78.9	1.2	4.8	1.5%	6.0%
Calling	Female	1	129	132	131	3.0%	2.0%	79.9	78.8	89.4	1.1	9.5	1.4%	12%
		2	139	137	137	1.7%	1.7%	79.9	79.1	89.8	0.8	9.9	1.0%	12.4%
	Male	1	125	125	125	0%	0%	79.9	79.7	74.1	0.2	5.8	0.3%	7.2%
		2	103	104	102	1.0%	0.9%	79.9	79.8	73.1	0.1	6.8	0.2%	8.6%
	Average					1.4%	1.2%	79.9	79.3	81.6	0.6	8.0	0.7%	10%
Swinging	Female	1	140	143	143	2.9%	2.8%	79.9	81.3	87.8	1.4	7.9	1.7%	9.9%
		2	129	130	137	1.0%	8.0%	79.9	81.8	86.8	1.9	6.9	2.4%	8.6%
	Male	1	130	132	132	1.6%	1.4%	79.9	81.6	86.8	1.7	6.9	2.1%	8.6%
		2	100	101	102	1.1%	2.0%	79.9	79.5	71.1	0.4	8.8	0.5%	11%
	Average					1.7%	3.5%	79.9	81.0	83.1	1.3	7.6	1.7%	9.5%

M1*: the results of the step length estimation considering the effective parameters. M2*: the results of the step length estimation without considering the effective parameters.

**Table 9:** Distance estimation on the 3D trajectory.

Mode	Path				Complex								
	Gender	Steps Count			Steps Count Error (%)		Distance (m)			Absolute Distance Error (m)		Relative Distance Error (%)	
		Actual	M1*	M2*	M1*	M2*	Actual	M1*	M2*	M1*	M2*	M1*	M2*
Reading	Female	216	214	222	0.9%	2.8%	105.2	104.6	140.6	0.6	35.4	0.6%	33.6%
	Male	162	166	171	2.5%	5.6%	105.2	104.3	121.5	0.9	16.3	0.8%	15.5%
	Average				1.7%	4.2%	105.2	104.5	131.0	0.7	25.8	0.7%	24.5%
Calling	Female	213	214	215	0.5%	0.9%	105.2	104.3	138.7	0.9	33.5	0.9%	31.8%
	Male	160	163	163	1.9%	1.9%	105.2	103.2	124.2	2.0	19.0	1.9%	18.1%
	Average				1.2%	1.4%	105.2	103.7	131.5	1.5	26.3	1.4%	25.0%
Swinging’	Female	216	219	210	1.4%	2.8%	105.2	106.3	133.1	1.1	27.9	1.0%	26.5%
	Male	180	178	173	1.1%	3.9%	105.2	104.0	127.4	1.2	22.2	1.2%	21.1%
	Average				1.3%	3.3%	105.2	105.1	130.2	1.1	25.0	1.1%	23.8%

M1*: the results of the step length estimation considering the effective parameters. M2*: the results of the step length estimation without considering the effective parameters.

**Table 10:** Positioning results of the texting mode.

Gender	Strategies	Subject	Distance Estimation		Final Positioning		CDF			
			Absolute Error (m)	Relative Error (%)	Absolute Error (m)	Relative Error (%)	Mean	STD	80%	95%
Female	Proposed	1	1.27	0.80	1.01	0.63	0.98	0.61	1.43	1.65
		2	1.52	0.96	1.61	1.01	1.58	0.91	2.21	2.77
		Average	1.40	0.88	1.31	0.82	1.28	0.76	1.82	2.21
	PDR+ activity Detection	1	6.28	3.94	1.63	1.02	1.41	1.30	1.78	2.59
		2	9.53	5.99	2.15	1.35	3.63	2.12	5.05	6.71
		Average	7.90	4.97	1.89	1.19	2.52	1.71	3.42	4.65
	PDR	1	28.20	17.71	1.65	1.04	1.26	2.88	3.81	4.44
		2	39.88	25.05	3.20	2.01	4.77	2.76	6.56	8.51
		Average	34.04	21.38	2.43	1.52	3.02	2.82	5.19	6.47
Male	Proposed	1	1.81	1.13	0.83	0.52	1.27	0.67	1.76	2.17
		2	1.86	1.17	1.42	0.89	1.25	0.69	1.70	2.21
		Average	1.83	1.15	1.13	0.71	1.26	0.68	1.73	2.19
	PDR+ activity Detection	1	21.50	13.50	1.45	0.91	4.63	2.17	6.03	7.11
		2	4.36	2.74	4.03	2.53	2.03	1.06	2.68	3.46
		Average	12.93	8.12	2.74	1.72	3.33	1.61	4.35	5.29
	PDR	1	46.55	29.24	1.19	0.75	3.66	1.69	4.74	5.26
		2	20.38	12.80	1.30	0.81	4.48	2.50	6.00	7.67
		Average	33.46	21.02	1.24	0.78	4.07	2.09	5.37	6.46

**Table 11:** Positioning results of the calling mode.

Gender	Strategies	Subject	Distance Estimation		Final Positioning		CDF			
			Absolute Error (m)	Relative Error (%)	Absolute Error (m)	Relative Error (%)	Mean	STD	80%	95%
Female	Proposed	1	1.28	0.81	1.69	1.06	1.35	0.67	1.75	2.44
		2	0.84	0.53	0.63	0.40	0.60	0.34	0.83	1.08
		Average	1.06	0.67	1.16	0.73	0.98	0.51	1.29	1.76
	PDR+ activity Detection	1	9.72	6.11	1.83	1.15	2.46	1.27	3.27	3.80
		2	3.67	2.30	1.14	0.71	1.10	0.62	1.49	1.92
		Average	6.70	4.21	1.49	0.93	1.78	0.94	2.38	2.86
	PDR	1	34.23	21.50	2.65	1.67	3.68	1.84	5.31	5.72
		2	17.66	11.09	2.92	1.83	3.35	1.68	4.43	5.73
		Average	25.94	16.30	2.79	1.75	3.52	1.76	4.87	5.72
Male	Proposed	1	2.17	1.36	0.51	0.32	0.87	0.58	1.39	1.69
		2	0.80	0.50	1.58	0.99	1.47	0.83	2.04	2.66
		Average	1.48	0.93	1.04	0.66	1.17	0.70	1.72	2.18
	PDR+ activity Detection	1	8.04	5.05	0.66	0.41	2.18	0.96	2.42	3.67
		2	5.04	3.17	4.17	2.62	2.80	1.28	3.52	4.52
		Average	6.54	4.11	2.41	1.52	2.49	1.12	2.97	4.10
	PDR	1	31.81	19.98	4.06	2.55	5.34	2.58	6.70	8.15
		2	9.09	5.71	2.47	1.55	3.11	2.03	4.40	5.91
		Average	20.45	12.84	3.27	2.05	4.23	2.31	5.55	7.03

**Table 12:** Positioning results of the swinging mode.

Gender	Strategies	Subject	Distance Estimation		Final Positioning		CDF			
			Absolute Error (m)	Relative Error (%)	Absolute Error (m)	Relative Error (%)	Mean	STD	80%	95%
Female	Proposed	1	1.63	1.02	0.86	0.54	1.24	0.51	1.64	1.85
		2	1.34	0.84	1.38	0.87	1.35	0.76	1.89	2.45
		Average	1.48	0.93	1.12	0.70	1.29	0.64	1.76	2.15
	PDR+ activity Detection	1	9.90	6.22	1.79	1.12	1.91	0.96	2.48	2.94
		2	8.45	5.31	3.47	2.18	2.39	1.54	3.30	4.35
		Average	9.17	5.76	2.63	1.65	2.15	1.25	2.89	3.64
	PDR	1	34.73	21.82	4.76	2.99	3.53	1.63	4.51	5.30
		2	20.64	12.96	3.25	2.04	4.09	2.31	5.27	6.76
		Average	27.69	17.39	4.00	2.52	3.81	1.97	4.89	6.03
Male	Proposed	1	1.41	0.88	1.37	0.86	0.93	0.50	1.30	1.62
		2	1.04	0.65	1.42	0.89	1.58	0.83	2.25	2.87
		Average	1.22	0.77	1.40	0.88	1.25	0.66	1.77	2.24
	PDR+ activity Detection	1	5.84	3.67	2.38	1.49	1.80	0.79	2.17	2.73
		2	5.99	3.76	3.29	2.07	3.24	1.95	4.47	5.90
		Average	5.91	3.71	2.84	1.78	2.52	1.37	3.32	4.32
	PDR	1	20.49	12.87	1.97	1.24	6.51	3.38	9.14	9.69
		2	17.51	11.00	6.00	3.77	5.22	3.67	7.66	9.41
		Average	19.00	11.93	3.99	2.51	5.87	3.53	8.40	9.55

**Table 13:** Comparison of positioning and distance errors of recent PDR methods.

		Gu et al., 2018 [18]	Klein et al., 2018 [16]	Wang et al., 2020 [17]	Lu et al., 2020 [43]	Geng et al., 2021 [14]	Park et al., 2021 [45]	Wu et al., 2021 [1]	Saadatzadeh et al., 2022 [56]	Proposed
Key parameters	Gender	Yes	No	No	Yes	No	No	No	No	Yes
	Height									
	Age									
	Walking speed	Yes	Yes	No	Yes	No	No	No	No	Yes
Mode	Items									
Texting	Distance error (m)	---	0.38	1.91	1.74	---	---		2.68	1.68
	Relative error (%)	---	1.8%	1.31%	1.74%	---	---		1.81%	1.05%
	Error at Final Position (m)	---	---	---	---	1.31	1.61	2.68	1.63	1.22
	Relative position error (%)	---	---	---	---	1.11%	2.77%	1.3%	1.1%	0.76%
Calling	Items									
	Distance error (m)	---	0.107	---	---	---	---	---	3.82	1.27
	Relative error (%)	---	0.5%	---	---	---	---	---	2.58%	0.8%
	Error at Final Position (m)	---	---	---	---	---	---	---	1.13	1.1
	Relative position error (%)	---	---	---	---	---	---	---	0.76%	0.69%
Swinging	Items									
	Distance error (m)	3.01	0.47	---	---	---	---	---	8.39	1.35
	Relative error (%)	3.01%	2.2%	---	---	---	---	---	5.65%	0.85%
	Error at Final Position (m)	---	---	---	---	---	3.94	---	1.68	1.26
	Relative position error (%)	---	---	---	---	---	6.79%	---	1.13%	0.79%
Experiment’s Length (m)		100	21.4	146	100	118	58	210	148.53	159.2

## Data Availability

Data sharing not applicable.
